# Supplementary material for: Deep learning-based feature extraction for prediction and interpretation of sharp-wave ripples in the rodent hippocampus
Source: eLife. 2022 Sep 5;11:e77772. doi: 10.7554/eLife.77772 (PMC9560163; doi:10.7554/eLife.77772)
Supplement: Supplementary file 1. [file elife-77772-supp1.docx]

**Table 1. Sessions and animals used for the different analysis**

Summary of the experimental sessions used for training and validation

| **Animal** | **Session** | **Duration (s)** | **#ripples** | |
| --- | --- | --- | --- | --- |
| **Training** | | | | |
| Amigo2_1 | hippo_2019-07-11_11-57-07_1150um | 2398.86 | 1309 | |
| Som_2 | hippo_2019-07-24_12-01-49_1530um | 1036.25 | 485 | |
| **Offline validation** | | | | |
| Thy7 | 2020-11-11_16-05-00 | 744.21 | 1064 | |
| Thy7 | 2020-11-11_16-21-15 | 763.67 | 926 | |
| Thy7 | 2020-11-11_16-35-43 | 701.99 | 656 | |
| Thy1GCam1 | 2020-12-18_13-16-03 | 708.92 | 301 | |
| Thy1GCam1 | 2020-12-18_13-32-27 | 669.15 | 412 | |
| Thy1GCam1 | 2020-12-18_14-40-16 | 613.75 | 245 | |
| Thy1GCam1 | 2020-12-18_14-56-54 | 725.4 | 237 | |
| Thy1GCam1 | 2020-12-21_14-58-51 | 630.61 | 115 | |
| Thy1GCam1 | 2020-12-21_15-11-32 | 651.16 | 159 | |
| Thy1GCam1 | 2020-12-21_15-26-01 | 682.22 | 165 | |
| Calb20 | 2021-01-22_13-08-20 | 1203.23 | 412 | |
| Dlx1 | 2021-02-12_12-24-56 | 1200.67 | 254 | |
| Dlx1 | 2021-02-12_12-46-54 | 1021.34 | 211 | |
| Thy9 | 2021-03-16_12-10-32 | 1516.65 | 264 | |
| Thy9 | 2021-03-16_14-31-51 | 1201.19 | 274 | |
| **Online validation** | | | | |
| PV6 | 2021-04-19_14-02-31 | 1051.75 | 422 | |
| PV7xChR2 | 2021-05-18_13-08-23 | 958.53 | 80 | |
| PV7xChR2 | 2021-05-18_13-24-33 | 855.42 | 121 | |
| PV7xChR2 | 2021-05-18_13-08-23 | 958.53 | 88 | |
| Thy10 | 2021-06-01_13-28-27 | 626.62 | 318 | |
| Thy10 | 2021-06-15_15-28-56 | 976.90 | 566 | |
| Som_8 | Som8_220209_123505 | 646.72 | 108 | |
| WT04 | WT04_220314_115332 | 601.63 | 211 | |
| **Neuropixels** | | | | |
| Calb | 28Jul_g0_imec0 | 754.28 | 166 | |
| Thy1 | 01Jul_g0_imec0 | 643.60 | 709 | |
| Thy1 | 15Jul_g0_imec0 | 690.95 | 124 | |
| Thy1 | 16Jun_g0_imec0 | 655.70 | 625 | |
| **External database** | | | | |
|  | | | **Original** | **Validated** |
| Achilles | Achilles_10252013 (first 30 min chunk) | 1800.00 | 223 | 423 |
| Achilles | Achilles_10252013 (fifth 30 min chunk) | 1800.00 | 470 | 821 |
| Achilles | Achilles_11012013 (first 30 min chunk) | 1800.00 | 569 | 283 |
| Cicero | Cicero_09012014 (first 30 min chunk) | 1800.00 | 264 | 133 |
| Cicero | Cicero_09012014 (third 30 min chunk) | 1800.00 | 515 | 743 |
